# Tweets Related to Motivation and Physical Activity for Obesity-Related Behavior Change: Descriptive Analysis

**DOI:** 10.2196/15055

**Published:** 2022-07-20

**Authors:** Albert Park

**Affiliations:** 1 Department of Software and Information Systems College of Computing and Informatics University of North Carolina-Charlotte Charlotte, NC United States

**Keywords:** obesity, motivation, exercise, peer support, social network analysis, social computing, consumer health information, informatics, information science, social support, communications media

## Abstract

**Background:**

Obesity is one of the greatest modern public health problems, due to the associated health and economic consequences. Decreased physical activity is one of the main societal changes driving the current obesity pandemic.

**Objective:**

Our goals are to fill a gap in the literature and study whether users organically utilize a social media platform, Twitter, for providing motivation. We examine the topics of messages and social network structures on Twitter. We discuss social media’s potential for providing peer support and then draw insights to inform the development of interventions for long-term health-related behavior change.

**Methods:**

We examined motivational messages related to physical activity on Twitter. First, we collected tweets related to physical activity. Second, we analyzed them using (1) a lexicon-based approach to extract and characterize motivation-related tweets, (2) a thematic analysis to examine common themes in retweets, and (3) topic models to understand prevalent factors concerning motivation and physical activity on Twitter. Third, we created 2 social networks to investigate organically arising peer-support network structures for sustaining physical activity and to form a deeper understanding of the feasibility of these networks in a real-world context.

**Results:**

We collected over 1.5 million physical activity–related tweets posted from August 30 to November 6, 2018. A relatively small percentage of the tweets mentioned the term *motivation*; many of these were made on Mondays or during morning or late morning hours. The analysis of retweets showed that the following three themes were commonly conveyed on the platform: (1) using a number of different types of motivation (self, process, consolation, mental, or quotes), (2) promoting individuals or groups, and (3) sharing or requesting information. Topic models revealed that many of these users were weightlifters or people trying to lose weight. Twitter users also naturally forged relations, even though 98.12% (2824/2878) of these users were in different physical locations.

**Conclusions:**

This study fills a knowledge gap on how individuals organically use social media to encourage and sustain physical activity. Elements related to peer support are found in the organic use of social media. Our findings suggest that geographical location is less important for providing peer support as long as the support provides motivation, despite users having few factors in common (eg, the weather) affecting their physical activity. This presents a unique opportunity to identify successful motivation-providing peer support groups in a large user base. However, further research on the effects in a real-world context, as well as additional design and usability features for improving user engagement, are warranted to develop a successful intervention counteracting the current obesity pandemic. This is especially important for young adults, the main user group for social media, as they develop lasting health-related behaviors.

## Introduction

Obesity is continually increasing around the world [1]. The annual economic cost of obesity is a substantial burden [2], because it is associated with type 2 diabetes, heart disease, stroke, arthritis, high blood pressure, and various cancers [1,3]. For example, total obesity-related medical expenditures are estimated at $116 billion in the United States [4]. Despite obesity’s serious health and economic consequences, two-thirds of US adults and nearly a third of children aged 6 to 19 years are either at risk for obesity or are obese [3,5]. Society-level behavior changes, including reduced physical activity, have been determined to be among the main contributors driving the current obesity pandemic [6]. To counteract this modern public health problem, we not only need to initiate society-level behavior changes (eg, increase physical activity), but also need to ensure adherence to the changed behaviors [7].

Studies have demonstrated long-term (ie, more than 18 months) exercise adherence challenges for obese individuals [8], even for individuals who had near-death experiences [9,10], due to issues such as dropout [11]. Peer support has been successful in sustaining changed behaviors, especially in the context of the Alcoholics Anonymous group [12,13]. Peer support has also been suggested to be more effective than financial incentives [14] or nurse care management [15] for people with diabetes. The concept has been successfully applied in mental health [16], acute substance abuse [17], diabetes control [14,15], and efforts to maintain physical activity [18]. However, not all peer support systems are successful [8]. One major contributing factor for developing an effective peer support system is to identify proper peer support pairs [19] or workers [16].

Social media has become an integral part of daily life in the United States [20]. Individuals use social media to freely express their thoughts and to interact with geographically dispersed like-minded individuals. Given the increasing use of social media, health-related research utilizing these platforms is also increasing. For instance, social media data have been applied in improving public health outcomes [21], conducting disease surveillance [22-24], enhancing personal health care experiences [19,25,26], examining the daily struggles of living with mental health conditions [27], predicting adverse drug reactions [28], and understanding user responses during a global pandemic [29]. Despite evidence suggesting that social media has the potential to influence individuals [30-37] and to identify exercise partners [38], we lack knowledge on how users naturally utilize social media for peer support, especially how social media is used to provide motivation for exercise adherence in the context of obesity and weight loss.

Many studies have examined social media or incorporated it into their design to investigate attempts to change health-related behaviors [30], such as by increasing physical activity [32-34], losing weight [35], and adhering to exercise programs [36,37]. However, substantial differences have been demonstrated in the use of social media, and these studies have not delivered long-term impacts in reducing the prevalence of obesity. Moreover, effective design elements are unknown; thus, many studies primarily focus on designing weight loss or weight management interventions that are delivered via social media [39-47], or they investigate relevant elements for higher user engagement [48]. To develop an effective intervention, it is imperative to first understand how users are organically utilizing the platform to provide or gain motivation and peer support, defined here as the exchange of motivational tweets for exercise in general, and then draw insights to inform the development of an intervention that provides peer support for long-term exercise adherence. In this study, we characterize the naturally occurring social network structures of peer support pairs or groups for motivating physical activity via a mixed methods approach. More specifically, we examine messages related to physical activity and motivation on a social media platform (Twitter). This study seeks to answer 2 research questions (RQs) to fill the gap in the literature. RQ1: Do Twitter users utilize the platform to provide or gain motivation for physical activity? RQ2: Do individuals use Twitter to form peer support pairs or groups for motivating physical activity? We restricted our analysis to publicly available discussion content.

## Methods

### Data Collection and Social Media Site

Twitter is a popular, free-to-use microblogging social media platform on which users can instantly broadcast short messages. These short messages are called tweets. Tweets are limited to 280 characters (this changed from 140 characters in November 2017) and are meant to be broadcasted to the world. Twitter users, however, can also send out directed, conversational messages by using the @ symbol followed by a Twitter user’s ID. Twitter does not have topically focused groups, but Twitter users can use the # symbol to label and categorize their tweets with searchable keywords. We employed the Python library Tweepy [49] and used hashtags from a previous study [50], including #weightloss, #diet, #fitness, and #health, to collect tweets related to physical activity and weight loss. We then extracted motivation-related tweets to answer RQ1 and reciprocal conversational interactions to answer RQ2. In this study, we only examined tweets in English.

### RQ1: Do Twitter Users Utilize the Platform to Provide or Gain Motivation for Physical Activity?

We first used a lexicon-based approach, which is a method to identify related text passages based on a set of words or phrases, to extract and examine motivation-related tweets from the overall physical activity tweets. A number of different motivation-related terms were considered, but for this study, we only used a single term, *motivation*, for the following three reasons. First, Twitter users frequently use acronyms and abbreviations due to the 280-character length limitation. However, in an online environment, these terms have been shown to change over time [51]. The same acronyms and abbreviations can also be used differently in different communities, which have specific cultures [52]. Second, we placed a higher priority on precision than recall in order to accurately observe user behavior and reduce noise. Third, many popular motivation-related hashtags contain the term *motivation* (eg, #fitnessmotivation and #mondaymotivation). Thus, we filtered for tweets that contained the keyword *motivation*. Specifically, we first preprocessed the entire data set to convert the text to lowercase. Then, to extract tweets containing our key terms from the entire data set, we employed a lexicon-based approach and extracted tweets and data from their “user” and “entities” fields, including user names, screen names, locations, timestamps, and user mentions. We extracted and included any partial matches in this process to cover a wide variation in terms, such as #mondaymotivation.

To understand temporal trends in motivation-related messages, we first analyzed the data by week and by time of day. In this analysis, we included data from between September 1, 2018, and November 2, 2018, because the tweets in our original data had an uneven number of days of the week and were collected at different starting and ending time points. The hours of the day were grouped into eight periods: (1) early morning (4:00 AM to 6:59 AM), (2) morning (7:00 AM to 9:59 AM), (3) late morning (10:00 AM to 12:59 PM), (4) afternoon (1:00 PM to 3:59 PM), (5) evening (4:00 PM to 6:59 PM), (6) late evening (7:00 PM to 9:59 PM), (7) night (10:00 PM to 12:59 AM), and (8) late night (1:00 AM to 3:59 AM).

To characterize and understand these messages, we first qualitatively examined retweets—reposted or forwarded tweets—related to motivation. Retweets are important in Twitter, because they have a higher chance of reaching a large number of users and can represent messages that embody the collective ideation of the platform [53]. Thus, we qualitatively analyzed the general themes of retweeted tweets following the thematic analysis process [54,55]. A prespecified threshold of at least 50 retweets was applied for a tweet to be analyzed, due to the size of the data set. All of the quotes in this paper have been deidentified and slightly modified to protect the privacy of users following the guidelines suggested by a previous paper [56].

To quantitatively expand the retweet analysis, we created a topic model using latent Dirichlet allocation (LDA) [57] to identify main topics from the larger data set. Topic modeling is an effective method to discover topics in a large data set and can identify a set of topics from a given set of documents based on document-level word co-occurrences. In our study, each tweet was considered a single document. First, we preprocessed the data set by removing URLs and tweets with fewer than 5 words. Similar exclusion criteria were applied to reduce noise in a previous study that used social media data [58]. Then, nouns were extracted using the Python natural language toolkit library [59] and used in the topic modeling process. LDA requires a predetermined number of topics, which can be a limitation. After experimenting with varying numbers of topics, we chose 10 topics related to motivation and physical activity on Twitter. An LDA topic model was generated by the Python library genism [60], followed by manual examination and labeling of the identified topics. To visually review these topics and occurrences of associated terms, we then visualized the topic model (ie, the main topics and their top 50 associated words) as a word cloud using the Python library wordcloud (Multimedia Appendix 1) [61]. Word cloud visualization is simple and user-friendly, yet scalable and preferred by users [62,63].

### RQ2: What Types of Reciprocal Conversational Social Network Structures Are Formed on Twitter for Motivating Physical Activity?

We generated 2 social network visualizations to verify whether social media enables organically formed peer support pairs or groups for motivating physical activity and to examine their social network structure. The first Twitter social network visualization represents a social structure of reciprocal, conversational interactions that are captured when users direct their tweets using the @ symbol to other Twitter IDs. These interactions are dyadic ties among Twitter users (determined by Twitter ID), who are the social actors. We first extracted all tweets directed toward other users by checking if the @ symbol was placed in conjunction with a Twitter ID in a tweet. We then use Gephi [64], a popular network visualization tool, to visualize the social network and apply the OpenOrd [65] layout to gain an overview of the network structure. The edge weight is determined by the number of interactions among Twitter users.

To examine Twitter’s real-world potential for connecting physical activity partners [38], we generated a second social network visualization of users who disclosed their location information. The purpose of this social network visualization was to visually examine the prevalence of social interactions that are generated from the same physical location and to assess the relationship between users’ current practices and the platform’s potential utility for connecting physical activity partners. Twitter users can opt to share their location information in their user profile. The Twitter API also provides a mechanism to track the geocode of a tweet; however, a previous study suggests that only 1.5% of tweets are geotagged [66]. Thus, we extracted users’ location information from their profile for the second social network visualization and also recorded the number of geotagged tweets and users who opted to disclose this information. We removed social actors without location information, and then estimated peer-supporting physical activity partners (ie, socially and physically connected users). We denoted this information using a coloring schema in the second social network visualization. We used the Fruchterman Reingold graph layout algorithm [67] for the second visualization, which had a network structure with considerably fewer social actors and dyadic ties.

### Ethics Statement

The study was determined to not have human subjects by the University of North Carolina-Charlotte's Institutional Review Board (Ethics Committee).

## Results

### Data Set

We collected over 1.5 million physical activity–related tweets made between August 30, 2018, and November 6, 2018. Table 1 summarizes the overall data set and the data sets for each research question. 

**Table 1:** Summary of data set.

Tweet type	Physical activity tweets (N=1,528,439), n (%)	Unique Twitter IDs (N=474,402), n (%)
Motivation (research question 1)	39,703 (2.6)	13,561 (2.86)
Directed motivation (research question 2)	15,720 (1.03)	6558 (1.38)

### RQ1: Do Twitter Users Utilize the Platform to Provide or Gain Motivation for Physical Activity?

#### Overview

Of the 1.5 million physical activity–related tweets, we found that 2.6% (39,703/1,528,439) contained the term *motivation*. This is a relatively small proportion of the overall physical activity–related tweets, but it still amounted to 39,703 tweets made by 13,561 unique Twitter IDs. Of these 39,703 motivational messages, the most were sent out on Mondays (7070 tweets) and during morning (5668 tweets) or late morning (5748 tweets) hours (7:00 AM to 9:59 AM and 10:00 AM to 12:59 PM, respectively). Other days had substantially fewer tweets (from Tuesday to Sunday, in order: 5278, 5487, 5374, 5492, 5819, and 5220 tweets). Similarly, after late morning, motivational messages gradually decreased until the morning of the next day (from afternoon to early morning of the next day, in chronological order: 5247, 5284, 4955, 4930, 3866, and 4042 tweets). Within the morning and late morning hours, the most tweets were sent around lunchtime, from 11:00 AM to 11:59 AM (1987 tweets), followed by the 7:00 AM to 7:59 AM period (1949 tweets). Specific topics related to these tweets are further examined and described via qualitative analysis of retweets and topic modeling of the entire motivation data set in the following section.

In our data set, we found that 13,268 of the 39,703 (33.42%) overall motivation tweets were retweets. These 13,268 retweets retweeted 5915 (44.58%) original tweets. Of these 5915 original tweets, 18 were retweeted more than a prespecified threshold of 50 times. These 18 tweets were retweeted 1562 times, comprising 11.77% of the total number of retweets. Qualitative analysis of these 18 tweets identified the following 3 themes: *motivation*, *promoting*, and *information*.

#### Providing Motivation (Self, Process, Consolation, Mental, Quotes)

*Providing motivation* was the most common theme. However, a number of different subthemes were identified within this theme, including *self-motivation, motivation for continuing the physical activity journey/process, self-consolation, mental aspects of motivation*, and *use of quotes*. We categorized 12 of the 18 retweets as motivation. The following quote is a slightly modified example of a motivation retweet: “Finding motivation to workout doesn’t need to be a difficult process....”

#### Promoting (Individuals or Groups)

We categorized 4 of the 18 retweets into the *promoting* theme. Two types of promoting were identified, *promoting individuals* and *promoting groups*, for physical activity–related matters. One promoting method was to tweet out the names of individuals; thus, we do not show those tweets. Another promoting method was done using the following format in our data set: “Follow us for...”

#### Information (Sharing or Requesting Information)

We categorized 2 of the 18 retweets into the *information* theme. In these retweets, Twitter users voluntarily shared health tips or health-related information. It is also important to note that some retweets could have been made to promote products or brands, a practice known as “astroturfing.” Astroturfing is when sponsors or organizations mask themselves as Twitter users to promote their products [68,69]. We categorized these tweets as *information* because of the subtle nature of astroturfing on Twitter and a lack of knowledge regarding the motives for sharing such information. The following quote is a modified example of information sharing: “The Truth About The...”

Our topic modeling analysis expanded the findings of the qualitative analysis of retweets. Topic modeling revealed that the *providing motivation* topic had different types. Users often revealed their personal motivation for physical activities, which included tweets with the topics of *appearance*, *weight loss*, *lifestyle*, *health, wealth, mental,* and *personal motivation*. We also identified *workout inspiration* and *daily motivation* as subtopics that were part of the *providing motivation* topic.

Topic modeling also identified topics related to sharing or requesting information, specifically information related to the *nutrition-diet* topic. Compared to the retweets in this category, the tweets contained a greater number of products, tips, and information. Additional topics were identified in this process, mainly different types of workouts, including the *muscular training* and *bodybuilding* topics, presumably used by weightlifters. Topic modeling, however, did not identify a topic related to *promoting*. A total of 10 topics were identified via topic modeling; Table 2 shows sample terms from each of the identified topics. Multimedia Appendix 1 summarizes and provides an overview of all the identified topics and their related terms.

**Table 2:** Ten topics and samples of associated terms found in motivational tweets identified via topic modeling.

Topic label	Selected sample terms
Workout inspiration	motivation, inspiration, fitness, love, goals, fitnessmotivation, workout, quotes, music, mood, determination, fitnessquotes
Appearance	fitbeauty, bikinibody, beautycoolgym, beautycoolmotivation, ass, erossensual, look, amazing, weightloss
Muscular training	focus, workout, exercise, muscle, body, power, coach, hardwork, mindset, grind, strength, nutrition, passion, trainer, strong, bodygoals
Bodybuilding	gym, motivation, bodybuildingmotivation, gymlife, tip, abs, men, bodybuilder, goal, women, protein, squats, physique
Weight loss	weightloss, weightlossmotivation, weightlossjourney, tips, facts, resources, weightlosstransformation, hotbodiedsnaps
Lifestyle	body, health, mind, lifestyle, healthyfood, enjoy, fitnessgirl, gymshark, pain, hope, momlife, bodytransformation, obesity
Nutrition-diet	nutrition, diet, healthy, health, food, fashion, truth, coffee, ketogenic, weightwatchers, travel, science, supplements, foods, deal
Health; wealth; mental	health, fitness, muscle, excuses, gold, pushpullgrind, dreams, goals wealth, mind, healthylife, money, grindout, business
Personal motivation	weight, cardio, bodybuilding, weightloss, fitmotivation, fitnessmodel, fitnessaddict, keto, hormones, strength, girls, beach, girl
Daily motivation	workout, mondaymotivation, fitnessmotivation, day, week, time, darebees, days, Tuesday, work, session, mondaymood, saturdaymotivation

### RQ2: What Types of Reciprocal Conversational Social Network Structures Are Formed on Twitter for Motivating Physical Activity?

The overall social network of directed tweets consisted of 14,028 dyadic ties among 10,889 social actors (determined by Twitter ID). It is important to note that some tweets contained multiple indications of where they directed their tweets, included self-mentioning, or were directed to Twitter IDs that were not found in our data set. This resulted in a greater number of social actors than the total number of Twitter IDs but a lesser number of dyadic ties than the total number of tweets shown in Table 1 and Table 3. Table 3 summarizes the directed tweets and the creators of those tweets (determined by Twitter ID) in our data set. About 68% (4459/6558) of the Twitter users disclosed their location in their profile; however, users rarely disclosed their exact location by providing geocode information. Similar to a previous study [66], our data set consisted of about 1% (94/6558) geotagged tweets and Twitter users, resulting in less than 100 users who opted to disclose their exact location. 

**Figure 1:**
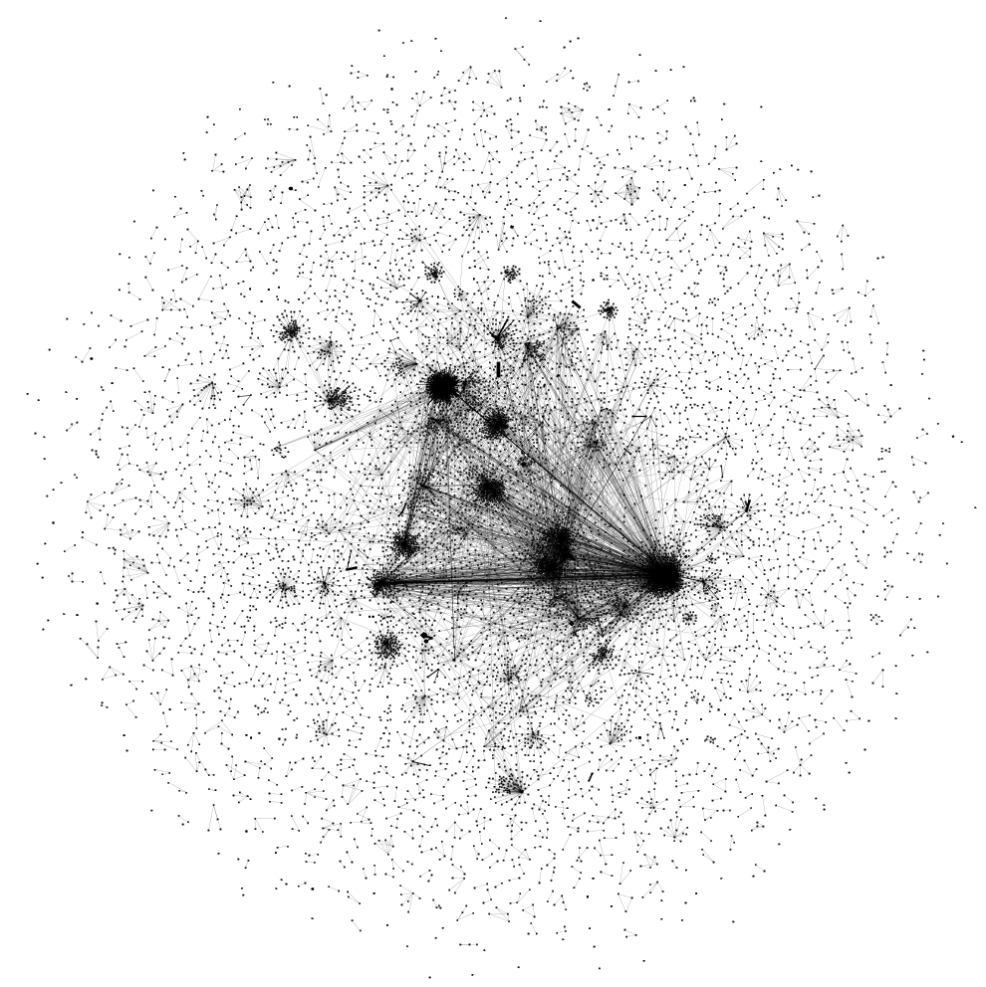
A social network of Twitter users sending directed tweets.

**Figure 2:**
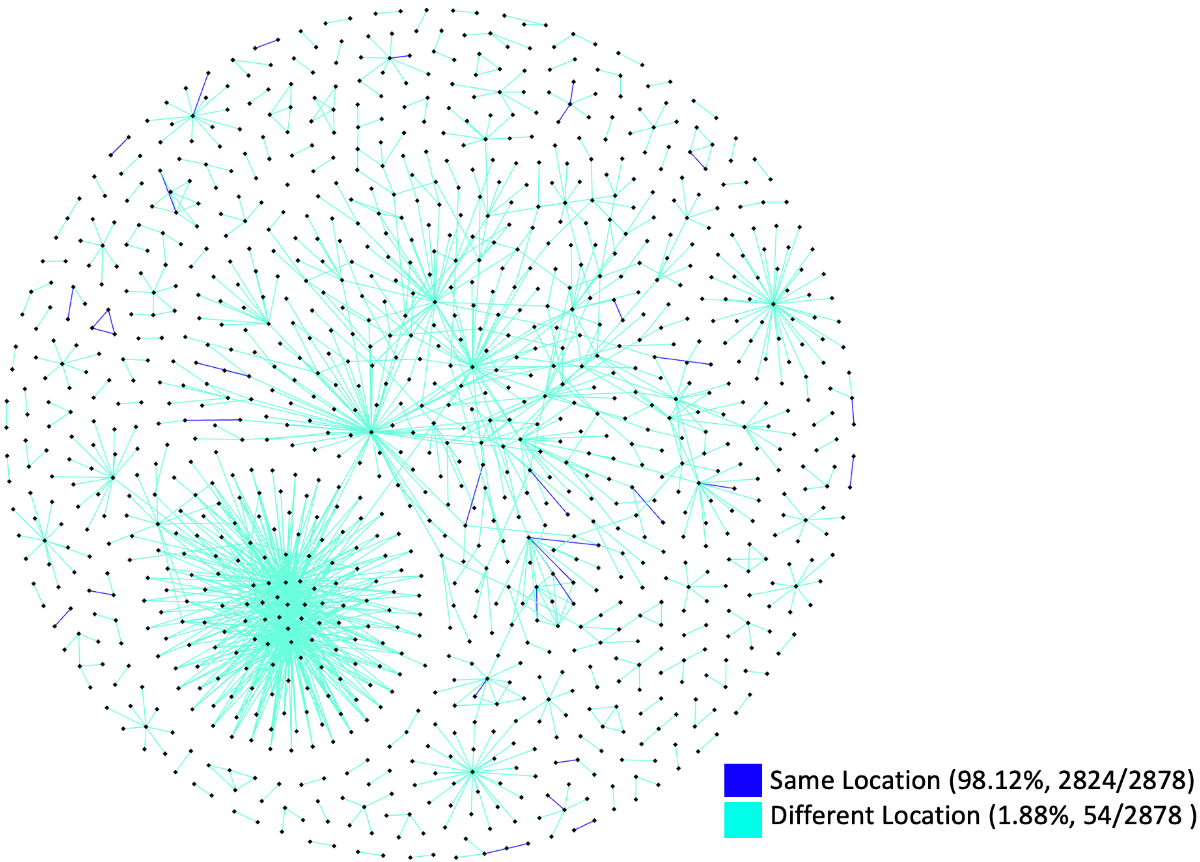
A social network of location-based Twitter user interactions. Twitter users from the same location are denoted in blue, whereas Twitter users from different locations are denoted in green.

Figure 1 shows an overview of the Twitter social network in our data set. Twitter users formed hundreds of small groups that sent motivation-related tweets to each other, while a small number of larger groups with a higher number of dyadic ties and social actors were formed. Figure 2 shows a social network of location-based Twitter user interactions. After removing social actors without location information, self-mentioning tweets, and interactions with only one-sided location information, the social network structure consisted of 2878 dyadic ties among 1112 social actors, a smaller number of social actors and dyadic ties than shown in Table 3. Of these 2878 social interactions, 1.88% (54/2878) were made from the same physical location.

**Table 3:** Summary of directed motivation tweet data set.

Tweet type	Total tweets (N=15,720), n (%)	Unique Twitter IDs (N=6558), n (%)
Directed at Twitter ID with location in the profile ID	10,533 (67)	4459 (67.99)
Directed tweet with geocode	159 (1.01)	94 (1.43)

## Discussion

This paper characterizes how Twitter users organically use a social media platform for motivating physical activity. To better understand Twitter’s potential as a long-term intervention platform, we specifically investigated and report elements that were related to peer support and motivation for physical activity. We also discuss the practical implications of our work and future directions.

### Practical Implications and Future Directions

#### Tweeting for Motivation

Twitter users use the platform to discuss and broadcast messages about motivation and physical activity. We extracted and analyzed a large number of motivation-related tweets (39,703 tweets from 13,561 unique Twitter IDs); however, these tweets were made by a small percentage of individuals (13,561/474,402, 2.86%) who tweeted about physical activity. Similarly, motivation-related tweets made up a small percentage (39,703/1,528,439, 2.6%) of overall physical activity–related tweets. In our extraction process, we purposely chose precision over recall, thus potentially reducing the overall number of motivation-related tweets. However, it is apparent that motivation was not the main focus of a large portion of the physical-activity tweets. A future study should identify other topics related to physical activity and examine how to further encourage and increase peer support for positive behavior change in social media. Despite this, messages by users to motivate themselves, as well as others, were typically sent on Mondays and in the morning and late afternoon hours. This could indicate that Twitter users need the most motivational support and exercise during these time periods. A hybrid peer-support intervention augmented with an automatic motivational feature may be able to automatically provide encouraging messages when motivation is most needed (eg, Mondays and during morning and late afternoon hours), if peer supporters cannot. A future study should investigate the effectiveness of a similar hybrid intervention to verify whether automatically generated messages can help sustain and encourage physical activity among users.

#### Usage

Among retweets, we identified the following 3 themes: *motivation*, *promoting*, and *information*. As individuals promote themselves on social media, they gain more influence over other users. The characteristics of health information provided by credentialed experts and individuals have been suggested to be different [70,71]. Capitalizing on the influence of these individuals could provide another avenue for changing health-related behavior. However, a better understanding of the role of these individuals and their messages is needed. For example, although individuals’ experience-based information provides many benefits, it could also be misleading without proper contextual information. Twitter, with its 280-character limit, could mislead many individuals, and intermediary monitoring of the quality of health information may thus be required in health-behavior interventions that use the Twitter platform. Another interesting finding was that Twitter users use the platform for physical activity self-motivation. The rationale behind this phenomenon is largely unknown, and this could be another mechanism for sustaining physical activity.

#### Main User Demographics

The topic model revealed new information about the users. For example, our model suggests that a large portion of these individuals were weightlifters (suggested by the *muscular training* and *bodybuilding* topics) or trying to lose weight (suggested by the *weight loss* and *nutrition-diet* topics). Our analysis also suggests that individuals need reminders and inspiration to stay physically active (suggested by the *workout inspiration*, *personal* *motivation*, and *daily motivation* topics). Other motivation-related factors for physical activity included lifestyle choices, appearance, health, and wealth.

#### Other Findings

Previous literature suggests that individuals do not want to share their problems using individually identifying social media accounts, such as on Facebook [72]. Our analysis revealed a few terms that described users’ physical ability with negative terminologies (eg, *grind* and *pain*), although they were not prevalent. Future work should further identify struggles and concerns that may be harmful for individuals or that can be improved through better design.

Another theme was sharing information voluntarily. Qualitative analysis of retweets led us to suspect that this theme may have arisen from astroturfing, but further analysis is warranted to confirm this observation. Despite the potential misuse of social media, we also found that Twitter users positively motivate themselves and other users by sharing quotes, comforting themselves and others, and discussing the process of their journey and mental aspects of staying physically active. These findings are design elements that need to be considered for developing a successful intervention using social media to increase physical activity.

#### Peer Support

Online peer support systems, in which peer-to-peer interactions can scale up to arbitrary values of n to n, hold promise for identifying successful peer support groups. Features for identifying physical activity partners exist on a number of online social health activity networks [38], which suggests the need and preference for peer support concerning physical activity. Forging long-lasting connections among a large user base remains a research challenge, although past work has suggested that written communication style and personality matching are important in the success of online peer support systems [19,73]. Despite these challenges, we found empirical evidence that Twitter users use the platform to motivate individuals from different physical locations (2824/2878, 98.12%) to increase their physical activity. However, we do not know how Twitter users organically created these peer-to-peer connections or whether these individuals already knew each other prior to Twitter communication.

Twitter users forge connections using the @ symbol and directly provide or gain motivation for achieving their physical activity goals. In our social network analysis, we verified the existence of a few large, highly interconnected groups as well as hundreds of small groups with fewer interactions. Investigating linguistic differences in topics and communication style and differences in personality matching between the large and small groups could show individuals’ preferences and needs for varying sizes of motivation groups. Because our study provides a snapshot of existing peer-to-peer interactions, we are uncertain how Twitter users form these interactions, find peer support partners, and join groups, and we do not know the long-term effects. Future work could ask Twitter users about their experiences related to forming and continuing peer support systems on Twitter by using surveys and interviews to gain a deeper understanding of long-term behavior changes via social media. Similarly, understanding the effects of strong and weak ties in large groups is important in utilizing social factors for this purpose.

Our findings suggest that Twitter has not been connecting local users and that Twitter users are more engaged with geographically dispersed individuals. Less than 2% (54/2878) of directed tweets were made from the same physical location. Our findings indicate that geographic location is of low importance in providing peer support; as long as the support provides motivation, it can succeed despite the peers having few common factors (eg, the weather) affecting their physical activity. This presents a unique opportunity to identify successful peer support groups that provide motivation in large user bases.

Online peer support systems were observed in our study, but we do not know their effects in a long-term, real-world context, which should be considered in related future work. For example, the effects of online peer support related to (1) fostering positive behavior change and (2) sustaining long-term engagement need to be further examined. A previous study suggests that higher engagement with a group leads to more weight loss [74], but sustaining continuous interaction is a prominent challenge for many online social groups [75-80]. Our analysis of retweets also highlights potential commercial influence, or astroturfing, in Twitter networks. The extent of astroturfing and its influence over social structures and user behavior are unconfirmed and warrant further investigation to control commercial and astroturfing effects.

#### User Privacy

Although Twitter data are public, and tweets are meant to be broadcasted to the world, we discovered consistent user behaviors aimed at protecting user privacy. For example, we found that almost all users opted out of disclosing geocode information (ie, their exact location), as shown in Table 3. Also, even though the majority of users opted to disclose their location in their profile, many did not provide granular information (the limitation section has further details). Research that uses public social media data, including this study, is typically granted exemption from review by Institutional Review Boards in the United States. User behavior in our study indicates that ethical considerations are still needed, especially with regard to privacy [81,82]. In this paper, we did not present user-identifiable information (eg, Twitter IDs) and modified the example quotations to protect user anonymity.

### Limitations

A relatively short timeframe and a lack of seasonal coverage are limitations of our study. Though we did not examine seasonal trends, it is possible that posting frequency could change with the season. Similarly, previous work has shown that social media usage can vary temporally and spatiotemporally [83]. The time period in our study may also have affected trends in posting. Our study time period included a change of seasons, from late summer to fall, which may have been associated with changes in activity and exercise. Similarly, we manually examined nearly 11.77% (1562/13,268) of the total retweets, but the number of retweeted original tweets in our qualitative analysis was relatively small (18/5915). Although we used a prespecified threshold to select original tweets to be qualitatively examined, investigating a larger number of original tweets could have led to more conclusive findings. Moreover, future studies should examine long-term engagement with content among group members to understand the formation, evolution, and collapse of peer networks for the purpose of encouraging physical activities.

Twitter has a diverse user group compared to many other social media platforms, although it is still used more by young adults [84]. This could have created a bias toward younger Twitter users, allowing us to identify particular topics of interest only to this group. We do not know how our findings would apply to other demographics or social media sites. Similarly, our analysis focused on English tweets, and our findings may not be generalizable to non–English speaking countries. Our findings, however, have significant implications for young adults, the main user group of social media, and the health of future society, as they develop lasting health-related behaviors.

Another selection bias was present in the location-based Twitter user interaction analysis (Figure 2). Although the majority of users opted to disclose their location information in their profile (4459/6558, 67.99%), this analysis was conducted with users who self-selected to disclose this information. In the same analysis, an additional limitation was discovered in our manual validation check. We believe that the different location figure (2824/2878, 98.12%) was inflated due to the granularity of information provided by users. For example, in our data set, we found a few users who had identified their location as “Earth” or “America.” Moreover, some users disclosed their information at the state level (eg, North Carolina) and some users disclosed this information at the city level (eg, Charlotte). Although it is possible that they such users were in fact at the same physical location, we were not able to determine this without additional information. In our study, they were thus treated as being in different locations.

In this study, we used hashtags that were identified in a previous study that collected tweets related to physical activity for the purpose of weight loss. However, we did not incorporate postprocessing to include a wider variation in physical activities. The collected tweets were then processed to extract motivation-related tweets for RQ1. Initial key terms are important in collecting a relevant data set, and different sets of terms could have resulted in different findings, especially related to the prevalence of these tweets. Similarly, for RQ1, we used a single keyword, *motivation*, to identify motivation-related tweets. While this approach can identify related tweets with high precision, we should in future extend these study results to retrieve a greater number of relevant messages by developing a classifier. Moreover, major differences, such as communication style, user demographic, and the platform’s intended purpose, exist among social media platforms. To better characterize the landscape of peer support and motivating messages for physical activity in social media, replicating a similar study on a different popular social media platform, such as Facebook or Reddit, is warranted.

### Conclusions

Peer support through social media could be a potential means of encouraging positive behavior changes and sustaining that changed behavior. This study examines whether individuals organically use social media to encourage and sustain physical activity. The organic exchange of motivational messages and peer support are found among Twitter users, although this support is more commonly used by individuals from different physical locations. Our findings suggest that geographical location is less important in providing peer support, as long as the support provides motivation, despite the users having few factors in common affecting their physical activity (eg, the weather). Based on existing interaction patterns, we consider that successful motivation-providing peer support groups or interventions could be delivered through social media. However, further research on the effects in a real-world context, as well as additional design and usability features improving user engagement, are warranted to develop a successful intervention to counteract the current obesity pandemic. This is especially important for young adults, the main user group of social media, as they develop lasting health-related behaviors.

## Appendix

Multimedia Appendix 1 A word cloud overview of topic modeling: visual representation of main topics and frequently occurring associated terms.

## Abbreviations

LDA: latent Dirichlet allocation.
