# Supplementary figures and images for: Tweets Related to Motivation and Physical Activity for Obesity-Related Behavior Change: Descriptive Analysis
Source: J Med Internet Res. 2022 Jul 20;24(7):e15055. doi: 10.2196/15055 (PMC9350819; doi:10.2196/15055)

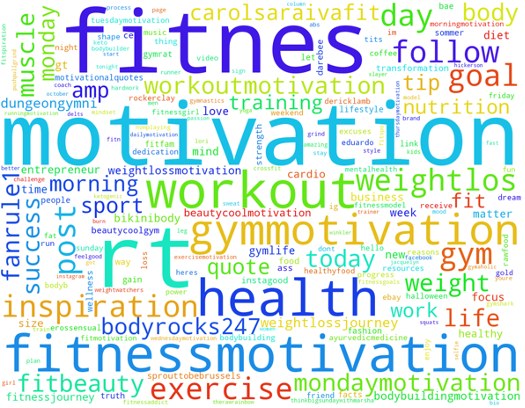

Supplement: Multimedia Appendix 1 [file jmir_v24i7e15055_app1.png]
